# Obesity‐related glomerulopathy: How it happens and future perspectives

**DOI:** 10.1111/dme.70042

**Published:** 2025-04-14

**Authors:** Jian‐Wen Samuel Lee‐Boey, Jia‐Kai Tan, Zhan‐Foong Lim, Francesco Zaccardi, Kamlesh Khunti, Majid Ezzati, Edward W. Gregg, Lee‐Ling Lim

**Affiliations:** ^1^ Department of Medicine, Faculty of Medicine Universiti Malaya Kuala Lumpur Malaysia; ^2^ Diabetes Research Centre University of Leicester, Leicester General Hospital Leicester UK; ^3^ Department of Epidemiology and Biostatistics, School of Public Health Imperial College London London UK; ^4^ School of Population Health Royal College of Surgeons of Ireland Dublin Ireland; ^5^ Department of Medicine and Therapeutics The Chinese University of Hong Kong Hong Kong SAR China; ^6^ Asia Diabetes Foundation Hong Kong SAR China; ^7^ Baker Heart and Diabetes Institute Melbourne Australia

**Keywords:** incretin therapies, nephropathy, obesity, pathophysiology, SGLT‐2 inhibitors

## Abstract

Obesity‐related glomerulopathy (ORG) is an emerging complication of excess adiposity. Its incidence rises alongside the obesity pandemic. Up to 40% of individuals can be affected by ORG, irrespective of the status of glomerular filtration rate and albuminuria. ORG is a distinct histological diagnosis based on kidney biopsy, showing classical features of an enlarged glomerulus with and without focal segmental glomerulosclerosis in the perihilar region seen with all categories of obesity. About 10% of individuals with ORG may progress to end‐stage kidney disease. The invasive nature of kidney biopsy highlights the need for non‐invasive biomarkers for improved screening, diagnosis and risk prediction of ORG. These biomarkers may narrow the gaps in the management of ORG by improving: (1) screening, diagnosis and differentiation of ORG from non‐ORG conditions; (2) risk prediction and stratification of individuals at risk of progression to end‐stage kidney disease including the detection of trajectories of progression; (3) monitoring of treatment safety and effectiveness and (4) development of novel therapeutic targets. In the present review, we discussed the pathophysiology, emerging biomarkers (such as kidney injury molecule‐1 [KIM‐1], uromodulin, klotho, circulating microRNA‐21 [miR‐21]) and future treatment strategies (metabolic surgery, sodium‐glucose cotransporter‐2 inhibitors, incretin‐based therapy and non‐steroidal mineralocorticoid antagonists) of ORG.

**What's new?**

- Up to one‐third of people with obesity‐related glomerulopathy (ORG) will develop a doubling of serum creatinine, and almost 10% will progress to end‐stage kidney disease.
- An increasing burden of obesity means more ORG cases may emerge; however, accurate identification and diagnosis require a kidney biopsy, which is an invasive procedure.
- Emerging blood‐ and urine‐based biomarkers such as kidney injury molecule‐1, uromodulin, klotho, proteomics CKD273, and transcriptomics microRNA‐21 may enhance diagnosis of ORG, but require further validation in people with obesity.

## 1 INTRODUCTION

**FIGURE 1:**
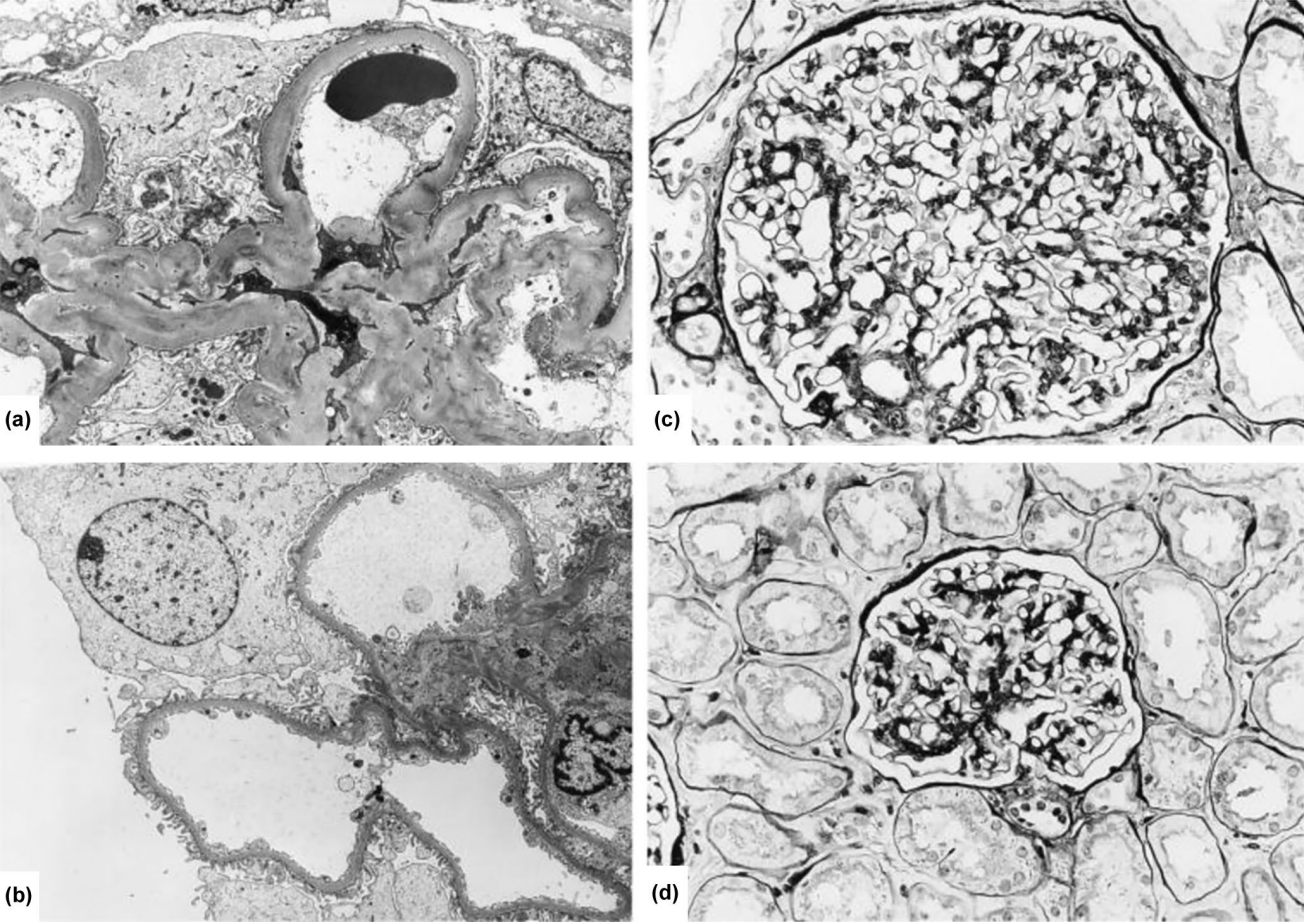
Representative electron microscopy (EM) and light microscopy (LM) findings in obesity‐related glomerulopathy (ORG). (a) EM image demonstrating thickening of the glomerular basement membrane and expansion of the mesangial matrix (“diabetoid” changes). (b) EM image highlighting mild podocyte foot process effacement typically observed in ORG. (c, d) LM illustrating characteristic glomerulomegaly and mild mesangial expansion in ORG, with the absence of significant segmental sclerosis lesions. Reprinted from Kidney International, Vol 59, Kambham N, Markowitz GS, Valeri AM, Lin J, D'Agati VD, “Obesity‐related glomerulopathy: An emerging epidemic,” Pages 1498–1509, Copyright (2001), with permission from Elsevier.

In the early 1970s, case studies observed a link between severe obesity and heavy proteinuria, leading to an entity called “obesity‐related glomerulopathy (ORG)”.^1^ ORG is diagnosed when there are (1) clinical obesity [body mass index (BMI) ≥30 kg/m^2^ and ≥25 kg/m^2^ for Western and Asian populations, respectively] and (2) kidney biopsy evidence of glomerulomegaly, with or without signs of focal segmental glomerular sclerosis (FSGS) (Table 1).^1^ While ORG shares similar pathological features with primary FSGS, it exhibits less severe microscopic changes such as foot process effacement <50% of glomerulus and fewer glomeruli with FSGS lesions (12% vs. 39%) (Figure 1 and Table 1).^2^

**TABLE 1:** Clinical and pathological differences between ORG and primary FSGS.

Feature	ORG	Primary FSGS	Key differentiating factors
Typical age at diagnosis	Older (mean 42.9 years)	Younger (mean 32.6 years)	ORG is linked to advancing age
Body mass index	Always ≥30 kg/m^2^ (mean 41.7 kg/m^2^)	Not necessarily associated with obesity	ORG is directly linked to obesity
Nephrotic syndrome	Rare (5.6%). Despite some have nephrotic‐range proteinuria (47.9%), they rarely present with the triad of nephrotic syndrome	Common (54%), often with edema	ORG rarely presents as full nephrotic syndrome
Serum albumin	Higher (mean 3.9 g/dL)	Lower (mean 2.9 g/dL)	ORG patients have better serum albumin levels
Edema	Less common (35%)	More common (68%)	ORG has milder fluid retention
Progression to kidney failure	Slower progression; 3.6% reached kidney failure (93 months)	Faster progression; 42% reached kidney failure (63 months)	ORG has a more indolent course
Histopathology (light microscopy)	Glomerulomegaly (100%), mild sclerosis	Minimal glomerulomegaly, segmental sclerosis (39%)	Glomerulomegaly is the hallmark of ORG
Foot Process Effacement (electron microscopy)	Less severe (~40%)	More severe (~75%)	ORG has milder podocyte injury
Response to RAAS blockade	Proteinuria improves (69.2%)	Variable response	ORG patients benefit more from RAAS inhibition
Response to steroids	Not routinely used	Standard treatment	Primary FSGS patients receive steroids more often
Association with metabolic syndrome	Strongly linked to obesity, diabetes, hypertension	FSGS more commonly associated with genetic, viral, drug‐induced	ORG is metabolically driven, while FSGS has multiple etiologies

*Note*: The numbers quoted in the figure are based on the landmark article by Kambham and colleagues in 2001.

Abbreviations: FSGS, focal‐segmental glomerulosclerosis; ORG, obesity‐related glomerulopathy; RAAS, renin‐angiotensin‐aldosterone system.

The identification of ORG is crucial, as up to one‐third of individuals with ORG will develop a doubling of serum creatinine, and almost 10% will progress to end‐stage kidney disease (ESKD).^1^, ^2^ Regarding progression to kidney failure, individuals with ORG lesions, whether alone or combined with other non‐ORG lesions, have a better prognosis than non‐ORG lesions alone (diabetic nephropathy, hypertensive nephrosclerosis, acute tubular necrosis, IgA nephropathy and minimal change disease).^2^, ^3^ Table S1 summarises the differences between ORG with diabetic and hypertensive nephropathy. Furthermore, ORG can occur in individuals with normal to subnephrotic range of proteinuria, with the latter potentially accelerating kidney dysfunction and mortality.

While the increased incidence in ORG parallels the obesity pandemic, not all individuals with obesity have ORG.^1^, ^2^, ^4^ Kidney biopsy studies reported that only up to 40% of individuals with obesity had ORG.^3^, ^5^ The identification of ORG requires a kidney biopsy, but this comes with procedural risks such as pain (4.3%), renal hematoma (11%), macroscopic haematuria (3.5%) and bleeding requiring blood transfusion (1.6%).^6^ The complexity of ORG driven by the intricate interplay of metabolic syndrome and insulin resistance, as well as the invasive nature of kidney biopsy, opens the avenues for non‐invasive biomarkers in providing further insights into disease mechanisms, enabling early screening, refining risk prediction, and serving as treatment monitoring tools. In the present review, we discuss the key pathophysiological features, as well as emerging biomarkers and treatment strategies related to ORG.

## 2 PATHOPHYSIOLOGY

### 2.1 Renal hemodynamic changes

Obesity significantly impacts kidney functions. Compared to those with normal weight, individuals with obesity have increased kidney plasma flow, glomerular filtration rate (GFR) and filtration fraction (calculated as GFR/kidney plasma flow). These changes are due to afferent arteriolar vasodilation and the relatively lower increment in kidney plasma flow than GFR.^1^ Cross‐sectional studies suggest that obesity is associated with an odds ratio (OR) of 2.28 (95% confidence interval [CI] 1.03–5.09) for glomerular hyperfiltration (defined as an eGFR >95th percentile after adjusting for sex, age, weight and height), with an OR of 3.52 (95% CI 1.79–6.91) for central obesity and OR 5.85 (95% CI 3.12–10.99) for general obesity.^7^ Furthermore, a cross‐sectional study demonstrated the single‐nephron GFR in 48 individuals with ORG and CKD (G1‐2) was significantly higher compared with the controls with and without obesity, providing evidence of single‐nephron hyperfiltration in humans with ORG and early stage of CKD.^8^ This suggests that glomerular hyperfiltration is a potential biomarker for early pathological damage of the kidneys.^9^ Future prospective studies and intervention trials are needed to confirm these findings and clinical implications among individuals with obesity.^10^

Compared to those with normal weight, individuals with obesity have higher sodium reabsorption at the proximal convoluted tubules due to glomerular hyperfiltration. Consequently, less sodium is delivered to the macula densa at the early distal convoluted tubule. This activates autoregulation via tubuloglomerular feedback, whereby the macula densa secretes vasoactive substances, causing afferent arteriolar vasodilation with increased GFR. This is supported by a randomised controlled trial (RCT) involving 12 individuals with obesity and glomerular hyperfiltration (but without diabetes), which compared the efficacy of acetazolamide (blocks 70% of sodium reabsorption at the proximal convoluted tubule) and furosemide (blocks 25% of sodium reabsorption at the ascending Loop of Henle).^11^ The acetazolamide group had an initial 21% decline in GFR due to amelioration of glomerular hyperfiltration, while the furosemide group had no decline in GFR. This can also potentially explain the cardiorenal benefits of sodium‐glucose cotransporter‐2 (SGLT2) inhibitors, which block sodium reabsorption proximal to the macula densa.^12^

### 2.2 Mechanical changes

Renal fat infiltration can lead to ORG. Excess visceral fat produces non‐esterified fatty acids (NEFAs), leading to increased hepatic production of very low‐density lipoprotein with their deposition at non‐adipose tissues (termed as “ectopic fat”). Ectopic fat deposition in the kidneys is associated with increased release of pro‐inflammatory factors and endoplasmic reticulum stress, worsening oxidative stress and insulin resistance. Most plasma fatty acids bound to albumin are reabsorbed by the proximal kidney tubular cells and stored as lipid droplets in the form of triglyceride. This ectopic deposition is commonly seen in obesity and can lead to lipotoxicity.^13^

Renal fat can be classified by its location: renal sinus fat, pararenal and perirenal fat and renal parenchymal fat. Renal sinus fat can mechanically compress the kidney vasculature, leading to increased hydrostatic pressure and reduced tubular outflow. This may lead to further activation of the renin‐angiotensin‐aldosterone system (RAAS) (Section 2.3). Excess fat accumulation may also lead to adipocyte hypertrophy and kidney hypoxia. Increased pararenal and perirenal fat in individuals with obesity can be detected from an ultrasound, computed tomography, or magnetic resonance imaging. Compared to those with normal weight, individuals with obesity have higher perirenal fat deposition. Notably, higher perirenal fat has been associated with suboptimal kidney outcomes in diverse patient groups including IgA nephropathy, type 2 diabetes, hypertension and heart failure.^14^

### 2.3 Neurohormonal changes

Individuals with obesity have RAAS overactivation, leading to increased sodium reabsorption at the distal convoluted tubule and collecting duct. Increased adiposity can also activate RAAS and worsen insulin resistance, leading to glomerular hyperfiltration. A weight loss of at least 5% has been shown to reduce the circulating levels of renin, angiotensinogen, aldosterone and angiotensin‐converting enzyme, which improves blood pressure control.

Adipose tissue can secrete adipocytokines such as adiponectin, leptin and resistin. Adiponectin inhibits the ROS/NF‐kB/NLRP3 pathway, exerting anti‐inflammatory and insulin‐sensitising effects. Notably, obesity is associated with reduced adiponectin release. In a model with adiponectin‐knockout mice, administration of adiponectin reduced podocyte permeability and podocyte dysfunction,^15^ as well as improved kidney inflammation in a model with obese rodents.^16^ Among 2238 Koreans with pre‐dialysis CKD, after a follow‐up of 3.5 years, serum adiponectin in the fourth quartile (16.80–79.88 μg/mL) was associated with a hazard ratio (HR) of 1.39 (95% CI 1.05–1.84) for a composite of kidney outcomes (defined as either a doubling of serum creatinine or the need for kidney replacement therapy), compared to those with serum adiponectin in the first quartile (≤5.09 μg/mL).^17^ Future studies are needed to validate the role of adiponectin as one of the emerging biomarkers for diagnosing and prognosticating ORG.

In a rat model without pre‐existing CKD, leptin infusion was associated with the development of glomerulosclerosis and proteinuria after 3 weeks, suggesting that leptin could upregulate surface TGF‐β2 receptors through signal transduction pathways.^18^ In a prospective study involving 2646 Korean adults without CKD,^19^ compared to serum leptin in the first tertile (male <1.69 ng/mL, female <6.08 ng/mL), serum leptin in the third tertile (male >2.91 ng/mL, female >9.89 ng/mL) was associated with an OR of 2.44 (95% CI 1.27–4.71) for incident CKD. However, this association was negated after adjusting for baseline eGFR. Furthermore, serum resistin may upregulate proinflammatory pathways via monocyte adhesion along the endothelium of blood vessels. High serum resistin is associated with suboptimal kidney outcomes in individuals with hypertension, diabetes and the elderly population.^20^ Taken together, both pre‐clinical and clinical studies support the key role of neurohormonal pathways in the development and progression of ORG.

### 2.4 Inflammatory changes

Obesity is increasingly recognised as a state of low‐grade chronic inflammation, characterised by increased macrophage infiltration and upregulated synthesis and release of pro‐inflammatory cytokines within adipose tissue. These inflammatory biomarkers include tumour necrosis factor‐α (TNF‐α), interleukin‐6 (IL‐6), interleukin‐1β (IL‐1β), C‐reactive protein (CRP) and monocyte chemoattractant protein 1 (MCP‐1).

TNF‐α enhances plasminogen activator inhibitor‐1 (PAI‐1) by mediating interaction between NF‐κB and a regulatory region of the PAI‐1 promoter. In turn, PAI‐1 directly enhances the infiltration and recruitment of pro‐fibrotic cells, leading to glomerulosclerosis and renal interstitial fibrosis. IL‐6 mediates insulin resistance by inhibiting insulin receptor signal transduction, promoting clot formation, and activating RAAS due to the upregulation of angiotensin II type 1 receptor gene expression. These mechanisms can worsen cellular injury within the kidneys. Pentoxifylline, a phosphodiesterase inhibitor, can reduce the synthesis of pro‐inflammatory cytokines such as IL‐1, IL‐6 and TNF‐α. Hence, two meta‐analyses reported the potential effects of pentoxifylline in slowing the decline in eGFR and reducing proteinuria.^21^, ^22^ We await more solid evidence from two ongoing RCTs, namely Pentoxifylline in Diabetic Kidney Disease (PTXRx) (NCT03625648) and PENFOSIDINE STUDY (Pentoxifylline Effect in Patients With Diabetic Nephropathy) (NCT03664414).

**FIGURE 2:**
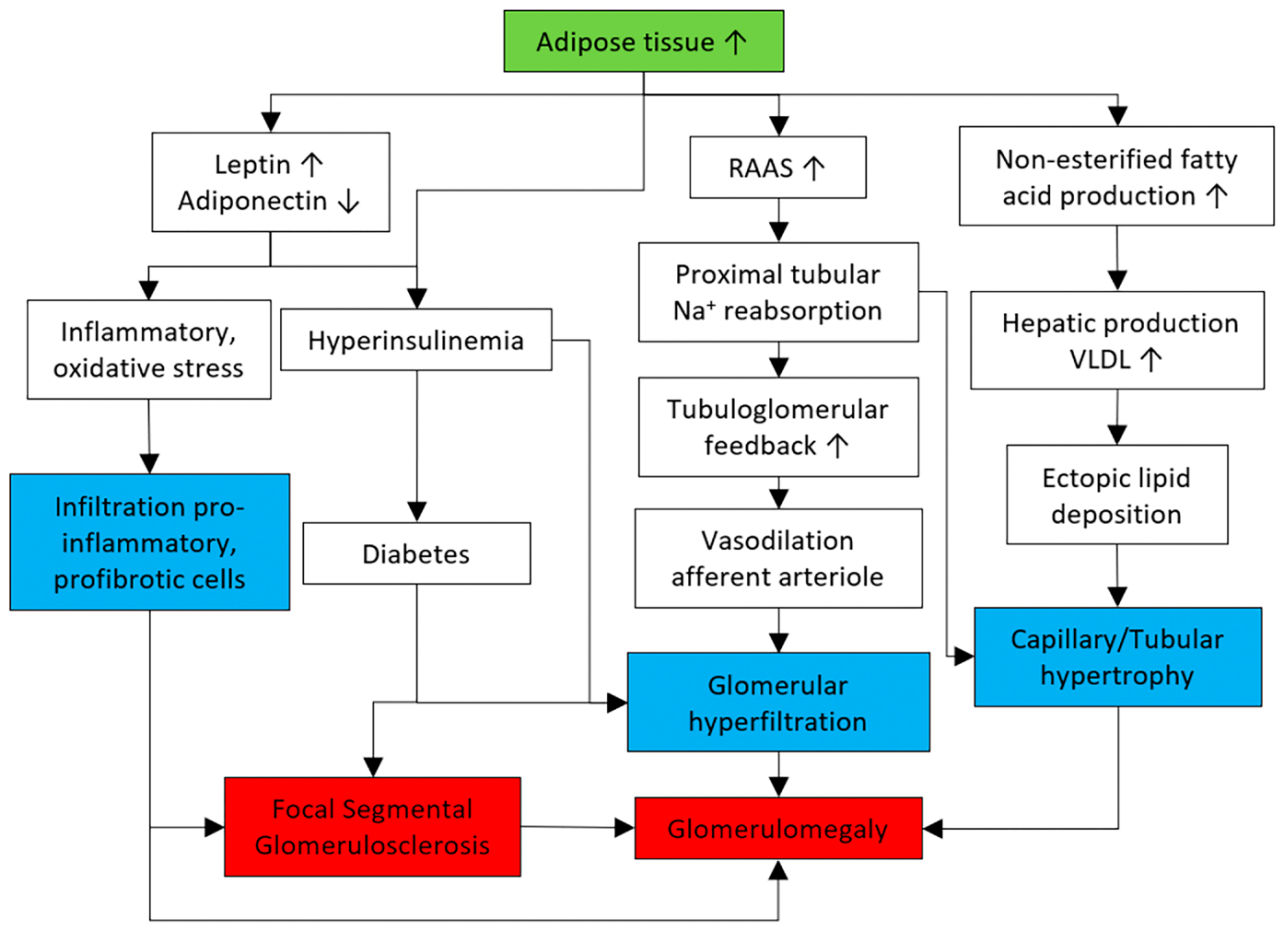
The overall mechanism of obesity‐related glomerulopathy. Boxes in blue colour refer to the pathological characteristic of obesity‐related glomerulopathy. RAAS, Renin‐Angiotensin‐Aldosterone system; VLDL, very‐low density lipoprotein.

A CKD‐induced rodent study found that a high level of CRP could contribute to early inflammation and fibrosis within the kidneys, likely due to the activation of both NF‐*κ*β and TGF‐β/Smad signaling pathways.^23^ MCP‐1 has also been suggested to directly elicit inflammation via cytokine and adhesion molecule expression in the kidneys, leading to tubulointerstitial inflammation. In a Phase 2 trial, semantical pegol (NOX‐E36), an MCP‐1 inhibitor with high affinity and specificity, reported a significant reduction of albuminuria by 29% after 3 months of treatment.^24^ Figure 2 summarises our current understanding of the mechanisms of ORG that can facilitate the discovery and validation of biomarkers that are useful for screening, detection, and treatment monitoring. The latter is discussed in Sections 3 and 4.

## 3 EMERGING BIOMARKERS

**FIGURE 3:**
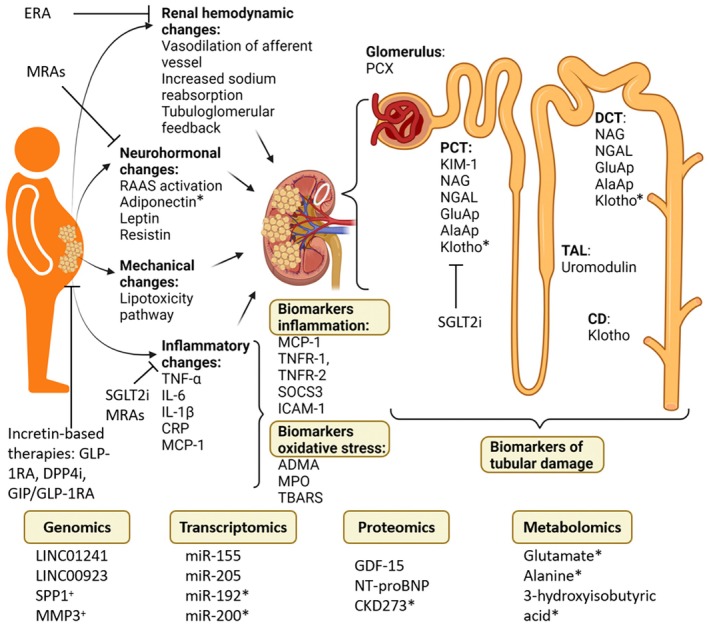
The mechanisms and candidate biomarkers in obesity‐related glomerulopathy. While many of the listed biomarkers show potential for chronic kidney disease (CKD) screening, only those marked with a cross (^+^) have been further explored for their role in risk prediction models, while biomarkers marked with an asterisk (*) have been investigated for their potential use in monitoring CKD treatment. ADMA, asymmetric dimethylarginine; CD, collecting duct; CRP, C‐reactive protein; DCT, distal convoluted tubule; DPP‐4i, dipeptidyl‐peptidase‐4 inhibitors; ERA, endothelin receptor antagonist; GDF‐15, growth differentiating factor‐15; GIP, glucose‐dependent insulinotropic polypeptide; GLP‐1RA, glucagon‐like peptide‐1 receptor agonists; ICAM‐1, intercellular adhesion molecule‐1; IL‐6, interleukin‐6; KIM‐1, kidney injury molecule‐1; MCP‐1, monocyte chemotactic protein‐1; MPO, myeloperoxidase; MRAs, mineralocorticoid receptor antagonists; NAG, *N*‐acetyl‐beta‐d‐glucosaminidase; NGAL, neutrophil gelatinase‐associated lipocalin; NT‐proBNP, N‐terminal pro‐B‐type natriuretic peptide; PCT, proximal convoluted tubule; PCX, podocalyxin; RAAS, renin‐angiotensin‐aldosterone system; SGLT2, sodium‐glucose cotransporter‐2 inhibitors; SOCS3, suppressors of cytokine signaling 3; TAL, thick ascending limb; TBARS, thiobarbituric acid‐reacting substance; TNFR‐1, tumour necrosis factor receptor‐1; TNF‐α, tumour necrosis factor α. Made with BioRender.com.

As obesity rates continue to rise globally, effective identification and management of ORG becomes increasingly important. Biomarkers hold promise as a non‐invasive and potentially cost‐effective alternative to kidney biopsy not only for diagnosing ORG, but also to guide decisions for the need for kidney biopsy in individuals with obesity. However, current evidence directly assessing biomarkers in individuals with biopsy‐proven ORG remains limited. Most available biomarker studies involve individuals with diabetic CKD, making it challenging to draw definitive conclusions for ORG per se. Nevertheless, given the overlap in pathogenic mechanisms between obesity and diabetes, insights from diabetic CKD studies may offer valuable preliminary evidence. Thus, findings from diabetes‐associated CKD studies are cautiously discussed here to highlight potential biomarkers relevant to ORG, acknowledging that further research specifically within the obese, non‐diabetic CKD populations is needed. Here, we discuss current and emerging biomarkers (Table S2) in the context of the underlying mechanisms of ORG as delineated in Section 2 and Figure 3.

### 3.1 Biomarkers of tubular damage

Uromodulin, initially known as the Tamm‐Horsfall protein, was first isolated from urine. It is a mucoprotein distributed along the cell lining of the thick ascending limb in the kidneys and reflects the remaining functional nephron cells. Compared to those without ORG, individuals with ORG exhibited a lower level of urinary uromodulin, reflecting a lack of compensatory response in the kidneys to increased metabolic demand from obesity. Observational studies reported in CKD populations that an elevated level of urinary uromodulin was associated with a reduced risk for a decline in eGFR and all‐cause mortality, showing an OR of 0.77 (95% CI 0.62–0.96) and 0.90 (95% CI 0.83–0.98), respectively.^25^

Neutrophil gelatinase‐associated lipocalin (NGAL) plays a role in embryonic nephron formation. It is rapidly induced in response to kidney failure. In a case–control study, individuals with obesity (but with normoglycaemia) had elevated levels of serum and urinary NGAL compared to healthy controls.^26^ Notably, serum NGAL levels increased with higher albuminuria levels. Kidney injury molecule‐1 (KIM‐1) is a member of the immunoglobulin gene superfamily, with its site of expression localised at proximal tubular epithelial cells. KIM‐1 increased with higher renal sinus fat deposition, potentially indicating ischemia and increased renal vasculature pressure due to excessive fat deposition within the kidney hilar regions. In type 2 diabetes populations, high levels of urinary KIM‐1 and NGAL are positively associated with albuminuria.^27^ A high plasma KIM‐1 has also been associated with an HR of 1.26 (95% CI 1.14–1.40) for a decline in eGFR among type 2 diabetes populations.^28^

*N*‐acetyl‐beta‐D‐glucosaminidase (NAG) is a lysosomal enzyme with renal proximal tubular origin. It is not filtered through the glomerulus due to its high molecular weight. Urinary NAG excretion was increased in the presence of microalbuminuria among individuals with diabetes.^29^ Podocalyxin (PCX), a type 1 transmembrane sialomucin protein under the CD34 family expressed in the apical surface of podocytes, forms the negatively charged barrier preventing protein loss into urine. A high level of urinary PCX was positively associated with the duration of obesity and albuminuria. Its sensitivity and specificity in detecting albuminuria were 83% and 74%, respectively, in the paediatric population.^30^ Further external validation studies with larger sample sizes are needed.

Klotho is a transmembrane protein expressed in the distal convoluted tubule, proximal convoluted tubule, and inner medullary collecting duct. It can inhibit TGF‐β, Wnt, and FGF2 signaling pathways and therefore mitigate kidney fibrosis. An elevated level of urinary Klotho may indicate damage to the proximal tubular lining with increased release of Klotho proteins into the luminal surface. In rat models with obesity, high levels of urinary Klotho, glutamyl aminopeptidase (GluAp), and alanyl aminopeptidase (AlaAp) were positively associated with increased proteinuria.^31^ In an observational study of 100 Chinese individuals, compared to healthy controls, individuals with ORG reported reduced levels of both serum and urinary Klotho, suggesting its role as a biomarker in identifying ORG.^32^ However, there is a need for a better understanding of the kinetics and standardisation of detection methods.

### 3.2 Biomarkers of Inflammation

Monocyte chemoattractant protein‐1 (MCP‐1) acts as a mediator of inflammation by regulating macrophage migration and infiltration. Compared to healthy controls, individuals with ORG reported increased levels of MCP‐1 and mast cells in kidney biopsies.^33^ However, it remains unclear whether mast cell infiltration is unique to ORG, necessitating further research. Also known as human cartilage glycoprotein 39 (HC‐gp39), YKL‐40 is a 40 kDa glycoprotein from a family of chitinases, with in‐vivo expression found in macrophages in different tissues. YKL‐40 can mediate inflammation and extracellular matrix remodeling. Urokinase‐type plasminogen activator receptor (uPAR) is a glycosyl‐phosphatidylinositol (GPI)‐linked membrane protein present on many types of immunologically active cells. When uPAR is cleaved, it gives rise to its soluble form (suPAR). Given its role in cell migration and adhesion during inflammation, serum suPAR increases when the immune system is triggered.

In the USA Chronic Renal Insufficiency Cohort (CRIC) cohort (*n* = 538; mean BMI 34 kg/m^2^), emerging biomarkers including plasma TNFR‐1, TNFR‐2, MCP‐1, suPAR and YKL‐40 reported inverse associations with eGFR, while having positive associations with proteinuria.^28^ Furthermore, a prospective study involving 410 American middle‐aged adults with diabetes (mean BMI 29.9 kg/m^2^) showed that high levels of plasma TNFR‐1 and TNFR‐2 at baseline were associated with an HR of 9.81 (95% CI 4.07–23.63) and 6.04 (95% CI 2.97–12.29) for ESKD, respectively.

The protein family of cytokine signaling (SOCS) suppressors is one of three classes of negative regulators for Janus kinase and signal transducer and activator of the transcription (JAK–STAT) pathway. In a study involving individuals with CKD G3–G4 (mean BMI 25 kg/m^2^), increased monocyte SOCS3 expression was associated with increased serum urea with a decline in eGFR.^34^ Intercellular adhesion molecule‐1 (ICAM‐1), an immunoglobulin‐like transmembrane glycoprotein with cell‐specific induction, regulates inflammation via innate and adaptive immunity. An increased level of serum ICAM‐1 was also associated with glomerular hyperfiltration in the paediatric populations.^35^

The nucleotide‐binding oligomerisation‐like receptor pyrin domain containing 3 (NLRP3) inflammasome is also implicated in ORG. Obesity can increase the level of angiotensin II, contributing to mitochondrial dysfunction, endoplasmic reticulum stress, and NLRP3 inflammasome pathway activation. These lead to the conversion of pro‐caspase 1 into active caspase 1 and upregulation of pro‐inflammatory cytokines such as IL‐1β and IL‐18. Notably, the administration of adiponectin has been shown to downregulate the NLRP3 inflammasome proteins and reduce kidney cell apoptosis and inflammation in rodent models.^36^ Taken together, these inflammatory biomarkers have shown preliminary evidence in ORG, which warrants further validations in clinical practice.

### 3.3 Biomarkers of oxidative stress

Increasing BMI is associated with worsening oxidative stress, enhanced cytokine production, and accelerated kidney injury. Asymmetric dimethylarginine (ADMA), an endogenous nitric oxide synthase (NOS) inhibitor, reduces the bioavailability of nitric oxide and contributes to endothelial dysfunction and afferent vasodilation in ORG. ADMA is metabolised by dimethylarginine dimethylaminohydrolase (DDAH), whereby its activity is impaired by high levels of oxidative stress. Among individuals with CKD and a mean BMI of 25 kg/m^2^, those with ADMA levels above the median cutoff had a 47% increased risk for CKD progression.

Myeloperoxidase (MPO), eosinophil peroxidase (EPO) and lactoperoxidase (LPO) make up a superfamily of mammalian haem peroxidase enzymes. MPO is mainly produced from neutrophils and can generate reactive oxygen species, leading to increased expression of adhesion molecules and glomerular injury. Among individuals with CKD and a mean BMI of 32 kg/m^2^, elevated plasma MPO was associated with an increased risk of CKD progression (defined as either ≥50% decline in eGFR, the need for kidney replacement therapy [KRT], or eGFR ≤15 mL/min/1.73 m^2^).^37^ Thiobarbituric acid reactive substances (TBARS) are often used as a marker of oxidative stress due to the cross‐reactions of thiobarbituric acid (TBA) with various aldehydes formed. This process may be enhanced during lipid peroxidation at the lipid‐rich membrane of renal tubules. Increased levels of plasma TBARS correlated with high BMI and a decline in eGFR.

### 3.4 Multi‐omics

Multi‐omics in the field of ORG is new. In a 2021 scoping review, 90 out of 123 included articles were focused on diabetes, whilst none were related to obesity per se.^38^ Hence, most hypotheses discussed in the following sections are based on the post‐hoc analyses of observational studies, either among the general population or individuals with diabetes.

#### 3.4.1 Genomics

Specific genetic mutations such as the apolipoprotein L1 (*APOL1*), non‐muscle myosin heavy chain 9 (*MYC*) and uromodulin (*UMOD*) are associated with an increased risk for CKD in the general population.^39^

Using the glomeruli samples of Chinese adults with biopsy‐proven ORG, a total of 256 genetic variants related to inflammatory cytokines (TNF‐α and its receptors, IL‐6 signal transducer, interferon‐γ and vascular endothelial growth factor [VEGF]), lipid metabolism (LDL receptor, SREBP‐1 and fatty acid binding protein 3) and insulin resistance (GLUT1, peroxisome proliferator‐activated receptor‐γ and leptin receptor) were identified. In the USA CRIC cohort,^40^ two single nucleotide polymorphisms (SNPs), namely *LINC00923 rs653747* and *rs931891*, were associated with a decline in eGFR. In the National Observatory of Atherosclerosis in Nephrology (NEFRONA) cohort, four SNPs, namely *SPP1* (Osteopontin) rs1126616, *MMP3* (matrix metalloprotease 3) *rs35068180*, *BGLAP* (Osteocalcin) *rs1800247* and *MGP* (Matrix Gla Protein) *rs4236*, were associated with a 29%–37% increased risk of CKD progression. By contrast, *CYP24A1 rs2248359* was associated with a 24% reduced risk of CKD progression. When these 5 SNPs were incorporated into a polygenic risk score and added to the conventional clinical risk factor model, the area under the curve (AUC) for detecting CKD improved from 0.794 (95% CI 0.770–0.819) to 0.824 (0.802–0.847). Given that Mendelian randomisation studies confirmed the causality between obesity and CKD, these findings further support the use of genomics to identify individuals with obesity who are at risk for the development and progression of ORG for timely interventions.^41^ Current genetic studies lack the diversity of populations and are mainly based on Western populations. Given the genetic heterogeneity and variations in population‐specific signals, it is essential to increase population diversity in genomics studies of ORG. Future research is also warranted to delineate the mechanistic pathways related to the novel genetic variants.

#### 3.4.2 Transcriptomics

Small RNAs are a distinct subclass of noncoding RNA and can be further classified based on their functions. Examples of small RNAs include microRNAs (miRNAs), transfer RNAs (tRNAs) and small nuclear RNAs (snRNAs). Small RNAs have a promising future because of their biofluid stability in serum, plasma, urine, and saliva. Since small RNAs are released from the cells via extracellular vesicles, they are also resistant to RNAse activity. Compared to lean controls, male obese rats showed significant associations of six urinary miRNAs (miR‐21‐5p, miR‐34a‐5p, miR‐192‐5p, miR‐29c‐3p, miR‐184, and miR‐132) with renal fibrosis. In a study involving 12 Korean adults (mean BMI 32 kg/m^2^), a high level of urinary snRNA U6 spliceosomal RNA (URS00006AAB7B) was associated with a decline in eGFR, whilst urinary hsa‐miR‐6124 had an inverse association with albuminuria. Compared to those without obesity, individuals with ORG reported several upregulated proteins using the single‐cell transcriptomic analysis performed on kidney biopsy, namely the SPP1 protein in T and B cells, podocin in podocytes, CCL2 and VCAM‐1in glomerular endothelial cells and insulin‐like growth factor‐binding protein‐7 in tubular epithelial cells.^42^ In a rodent model with diet‐induced obesity, upregulation of miR‐205 inhibited PTEN gene and linked to podocyte lipid endocytosis, an early manifestation of ORG.^43^ Taken together, miR‐21, miR‐29, miR‐6124 and miR‐205 are the emerging biomarkers for ORG, calling for more research to validate and evaluate their clinical utility.

#### 3.4.3 Proteomics

Compared to plasma/serum samples, urine samples are more chemically stable, easier for sample collection and non‐invasive. One significant protein in this context is Growth Differentiating Factor‐15 (GDF‐15), a cytokine from the TGF‐β family, which is secreted in response to cellular stress. An increased level of either serum or urinary GDF‐15 indicates inflammation, apoptosis and kidney fibrosis. In a 2023 meta‐analysis,^44^ individuals with the highest quartile of circulating GDF‐15 reported an HR of 2.60 (95% CI 2.06–3.27) for a composite of kidney endpoints (defined as either incident ESKD or ≥30% decline in eGFR), compared to those in the lowest quartile. Notably, this meta‐analysis was based on a limited dataset comprising only three studies of either plasma or serum measurements.

Another potential biomarker is the N‐terminal pro‐B‐type natriuretic peptide (NT‐proBNP), which is widely used to assess cardiac function. It can reduce blood pressure and alleviate heart strain through natriuresis, systemic vasodilation, and RAAS inhibition. In ORG, increased compression on kidney vasculature due to ectopic adipose tissue, leading to venous congestion, impaired kidney plasma flow, and chronic neurohormonal activation, potentially promotes secretion of NT‐proBNP. In a post‐hoc analysis of the Systolic Blood Pressure Intervention Trial (SPRINT), either a high level of serum NT‐proBNP at baseline or a 25% increase in NT‐proBNP level after 1 year was significantly associated with a ≥30% decline in eGFR.

Given that some proteins may carry low utility in diagnosis, they can be combined into a risk prediction model for cumulative effects. One of the examples is the urinary CKD273 proteomic classifier, which comprises collagen fragments, fibrinogen, apolipoproteins, hemoglobin, and alpha‐1 antitrypsin. Accumulation of collagen fragments indicates kidney fibrosis. Studies have also validated this classifier with 40%–75% sensitivity and 78%–79% specificity for incident CKD among the general population and individuals with diabetes.^45^ Notably, the role of urinary CKD273 in ORG is yet to be determined. A case–control study developed another classifier which comprised 150 collagen fragments (termed CKD150) that could identify individuals with obesity (BMI ≥30 kg/m^2^) who were at risk for CKD (defined as eGFR <45 mL/min/1.73 m^2^). However, only 79 adult cases with CKD were analysed and therefore further external validation studies of the proposed CKD150 are required. To date, proteomics studies are costly, with the estimated cost of each kit at USD 930.^45^ The increasing demand for proteomic studies may lower costs and spur more impactful research globally.

#### 3.4.4 Metabolomics

Metabolites can represent the summative effect of downstream genetic and protein expression and interactions with the environment. Several observational studies and meta‐analyses have demonstrated their predictive abilities for cardiovascular outcomes, but not mortality.^46^ Nevertheless, this method has several drawbacks, including the uniformity of sample collection, confounding factors (such as exogenous interference from bacteria and drugs), and challenges in identifying low‐abundance metabolites that may be associated with novel pathways.^47^ In the African‐American Study of Kidney Disease (AASK) and Modification of Diet in Renal Disease (MDRD) study involving 1582 adults with CKD at baseline (mean BMI 29 kg/m^2^), 58 serum metabolites (including 4‐hydroxychlorothalonil and 1,5‐AG) showed differential associations with proteinuria at baseline. After 7.9 years of follow‐up, only four (4‐acetamidobutanoate, N6‐carbamoylthreonyladenosine, N2,N5‐diacetylornithine, and Kynurenate) showed an increased risk of 38%–107% for incident ESKD, independent of proteinuria at baseline.

In a prospective study, 71 lipid and 23 protein metabolites were significantly associated with severe obesity and CKD, compared to severe obesity alone. The top three lipid metabolites were lysophosphatidylcholine (18:0), lysophosphatidylcholine (20:3), and phosphatidylcholine (35:3); while protein metabolites were isoleucine and tyrosine. In the CRIC cohort involving 1773 individuals with CKD (mean baseline BMI 32.2 kg/m^2^), three serum metabolites were significantly associated with incident ESKD and/or a ≥50% decline in eGFR, independent of eGFR and proteinuria at baseline.^48^ These results were replicated in the AASK and Hypertension and Atherosclerosis Risk in Communities studies.^48^ The metabolites of interest in ORG are pseudouridine (potentially a new filtration marker), methylimidazoleacetate (end‐product of histamine) and homocitrulline (protein carbamylation), whereby further external validation and clinical utility are needed. Future studies can also focus on examining the clinical, epigenetic, and metabolomic profiles of different subtypes of ORG such as with or without FSGS. Such classifications may facilitate a more personalised approach in its diagnosis and treatment strategies.

## 4 CURRENT AND FUTURE TREATMENT STRATEGIES

The identification and validation of the aforementioned biomarkers, rooted in the mechanistic understanding of ORG, provide a critical basis for developing and optimising targeted treatment strategies (Figure 3).

### 4.1 Lifestyle and metabolic surgery

Dietary and physical activity interventions play a pivotal role in preventing ORG. Although several meta‐analyses confirmed their efficacy in reducing albuminuria, most studies have included low sample sizes of <1000 individuals.^49^ Among 63 individuals with biopsy‐proven ORG who underwent a physician‐supervised diet and exercise intervention, at 2 years of follow‐up, individuals who achieved >3% reduction in BMI reported a 51% reduction in proteinuria and improved metabolic parameters (serum triglyceride, TG/HDL ratio, and blood pressure).^50^ In comparison, those who gained >3% BMI had a 28% increase in proteinuria. This suggests the benefits of weight loss on kidney functions in individuals with ORG.

Metabolic surgery is one of the most effective strategies for achieving substantial and sustainable weight loss with long‐term remission of underlying metabolic conditions. It can also reduce pro‐inflammatory cytokine levels. In a meta‐analysis of six observational studies,^51^ individuals with CKD G3 post‐metabolic surgery (via Roux‐en‐Y gastric bypass or laparoscopic sleeve gastrectomy) had improved GFR with a mean difference of 11.64 mL/min/1.73 m^2^ (95% CI 5.84–17.45), compared to pre‐metabolic surgery. Furthermore, long‐term studies support the beneficial effects of metabolic surgery on preserving kidney functions and slowing the decline in eGFR. In a prospective study among 92 adults with biopsy‐confirmed ORG, creatinine clearance levels remained stable at 2 and 5 years of follow‐up after metabolic surgery.^52^ Recent research has also highlighted the potential of multi‐omics such as urinary transcriptomics (miR‐192 and miR‐200) and serum/urinary metabolomics (lysine, threonine, proline, serine, valine, and glutamine) in monitoring the efficacy of individuals indicated for metabolic surgery.^53^, ^54^

### 4.2 Pharmacological therapy

Several recent RCTs have reported the cardiovascular‐kidney benefits of specific pharmacological therapies among individuals with type 2 diabetes and/or obesity. These have been discussed extensively in a recent review.^12^ Here, we discuss potential biomarkers that may act as novel treatment and monitoring tools for individuals with obesity and CKD.

#### 4.2.1 Sodium‐glucose cotransporter‐2 inhibitors (SGLT2i)

SGLT2i (canagliflozin, dapagliflozin, empagliflozin, ertugliflozin) increase sodium delivery to the macula densa and stimulate compensatory afferent arteriolar vasoconstriction to attenuate glomerular hyperfiltration. Multinational RCTs have reported that SGLT2i reduce the risk of cardiovascular and kidney outcomes in patients with either type 2 diabetes, heart failure, or CKD. In a prospective study involving middle‐aged Spanish adults with diabetes but without CKD, after 4 months, 45 adults who were treated with any SGLT2i had a significant increase in serum (5.2%) and urinary Klotho (38.9%) from baseline, as well as a significant reduction in urinary TNF‐α (23.8%, *p* < 0.01).^55^ Furthermore, the benefits of SGLT2i in individuals with non‐diabetic CKD (mean BMI 29 kg/m^2^) have been reported in the DAPA‐CKD and EMPA‐KIDNEY trials, showing an HR of 0.50 (95% CI 0.35–0.72) and HR 0.82 (95% CI 0.68–0.99), respectively, for a composite of kidney outcomes.^12^

Proteomics studies have also demonstrated its role in discovering novel therapeutic targets and monitoring tools. In a 12‐week RCT involving 32 Danish adults with type 2 diabetes and mean baseline uACR 154 mg/g (mean baseline BMI 33.7 kg/m^2^),^56^ the dapagliflozin‐treated group had improved CKD273 score (−0.221; 95% CI −0.356 to −0.087), albuminuria (−32%; 95% CI −48 to −9), and HbA_1c_ (−0.7%; 95% CI −1.0 to −0.4), compared to the placebo group. In another RCT involving 40 Danish adults (mean baseline BMI 25.8 kg/m^2^) with type 2 diabetes, albuminuria, and receiving RAAS, 36 urinary peptide fragments (relating to kidney fibrosis and function [type 1 and 3 collagens and albumin], inflammation [TGF‐β signaling], and wound healing [HSP90AB1 fragments]) were significantly altered after 12 weeks of dapagliflozin, compared to placebo.^57^

Compared to normal mice, obese mice had higher levels of metabolites related to phospholipid, purine, and biliverdin metabolism, along with a lower level of adenosine that was associated with tubuloglomerular feedback mechanisms.^58^ Notably, empagliflozin could normalise these metabolite changes. In an RCT involving 76 Chinese adults with diabetes, the empagliflozin‐treated group had a significant increase in serum adiponectin (2.8–3.6 mg/L; *p* < 0.0001) and plasma sphingomyelin, along with reduced glycochenodeoxycholate, cis‐aconitate, and uric acid levels, compared to the metformin‐treated group.^59^

#### 4.2.2 Incretin‐based therapies

Incretin‐based therapies include dipeptidyl‐peptidase‐4 (DPP4) inhibitors (such as linagliptin, saxagliptin, sitagliptin, vildagliptin), glucagon‐like peptide‐1 (GLP‐1) receptor agonists (such as exenatide, liraglutide, semaglutide), and dual glucose‐dependent insulinotropic polypeptide (GIP)/GLP‐1 receptor agonists (tirzepatide). A recent meta‐analysis of RCTs involving individuals with type 2 diabetes reported that the use of GLP‐1 receptor agonists was associated with a 21% risk reduction of a composite of kidney outcomes, compared to placebo.^60^ Furthermore, the SELECT trial, which involved 17,604 individuals with either overweight or obesity and established CVD but without diabetes, showed that a 2.4 mg weekly injection of semaglutide reduced the risk of kidney disease by 22%.^61^ Several metabolomics studies in obesity‐induced mice have uncovered potential kidney protective pathways associated with GLP‐1 receptor agonists. These include the upregulation of cardiolipin levels, reduced expression of pro‐inflammatory cytokines, and downregulation of insulin resistance. In diet‐induced obese mice,^62^ tirzepatide reduced branched‐chain amino acids (BCAA) and branched‐chain keto acids levels in brown adipose tissues, with an increase in BCAA end‐products such as glutamate, alanine and 3‐hydroxyisobutyric acid. Furthermore, the inflammatory process in ORG, characterised by mast cell infiltration and high MCP‐1 expression, was attenuated in liraglutide‐treated rodent models.^63^ Further research can assist in determining the role of these metabolites as potential treatment monitoring targets.

#### 4.2.3 Mineralocorticoid receptor antagonists (MRAs) and Endothelin receptor antagonists (ERAs)

Steroidal (spironolactone) or non‐steroidal (finerenone) MRAs suppress aldosterone and can slow CKD progression. The FIDELITY pooled analysis reported that the use of finerenone was associated with a 23% risk reduction of a composite of kidney outcome in individuals with type 2 diabetes and CKD, compared to placebo. A post‐hoc analysis of an RCT was performed in 111 adults with diabetes, hypertension, and receiving RAAS inhibitors.^64^ After 16 weeks of follow‐up, those who received spironolactone and had a higher baseline CKD 273 proteomic classifier score reported a significant decrease in uACR (β = −0.70, *p* = 0.049). The placebo group did not observe this effect (β = 0.39, *p* = 0.25). This suggests the role of CKD273 as a prognostic factor for CKD progression. In another post‐hoc analysis of an RCT involving 102 Danish adults with diabetes and hypertension, the spironolactone‐treated group with the lowest tertile of urinary metabolite score (related to inflammation and fibrosis processes) reported the highest uACR reduction (−54%), compared to the second (−41%) and the highest tertile (−17%; *p* = 0.01 for trend).^65^ A Japanese trial (NCT05887817) is ongoing to determine the effects of finerenone and related proteomic changes.

On the other hand, the SONAR trial reported the kidney protective benefits of atrasentan, an ERA, among 2648 adults with type 2 diabetes with eGFR 25–75 mL/min/1.73 m^2^, uACR 300–5000 mg/g and on maximally tolerated RAAS inhibitors (mean BMI 30 kg/m^2^).^66^ Compared to the placebo, the atrasentan‐treated group reported an HR of 0.65 (95% CI 0.49–0.88) for a composite of kidney outcomes (defined as a doubling of serum creatinine or sustained eGFR <15 mL/min per 1.73 m^2^, dialysis, kidney transplantation or death from kidney failure).

Control of multiple cardiometabolic risk factors, lifestyle modifications, metabolic surgery and certain pharmacological therapies have shown promising effects in treating ORG.^12^, ^67^ Given the growing landscape in this field, continued research is essential to unravel the complexities of ORG including its mechanisms, the discovery and validation of biomarkers and the advancement of treatment options.

## 5 CONCLUSION

A rising burden of obesity leads to multiple complications, notably ORG. This review provides an up‐to‐date comprehension of ORG's pathophysiology, which opens new avenues for clinical and translational research for improving health outcomes. An explicit understanding of the mechanistic pathways for discovering novel biomarkers can enhance the screening, monitoring, prevention and treatment of individuals with obesity, who are at risk of the development and progression of ORG. These person‐centric approaches, when integrated with the current standard of care, will reduce the disease burden and healthcare costs in the long run.

## AUTHOR CONTRIBUTIONS

LLL conceptualised the work. LBJWS, JKT, and LZF were involved in the literature review and drafting of the manuscript, while LLL finalised the manuscript. All authors participated in the data interpretation, revised the manuscript critically for important intellectual content, and approved the final version of the manuscript for publication.

## FUNDING INFORMATION

This study was funded by the UK Medical Research Council (Grant number MR/V034057/1; IF048‐2022). For the purpose of open access, the author has applied a Creative Commons Attribution (CC BY) license to the Author Accepted Manuscript version arising from this submission. KK is supported by the National Institute for Health Research (NIHR) Applied Research Collaboration East Midlands (ARC EM) and the NIHR Leicester Biomedical Research Centre (BRC).

## CONFLICT OF INTEREST STATEMENT

K.K.'s institution has received research grants from Boehringer Ingelheim, AstraZeneca, Novartis, Novo Nordisk, Sanofi, Lilly, and Merck Sharp & Dohme. He is a consultant to Novo Nordisk, AstraZeneca, Sanofi, Servier, Merck Sharp & Dohme, Novartis, Abbott, Amgen, Bayer, Lilly, Roche, Berlin‐Chemie AG/Menarini Group, and Boehringer Ingelheim. FZ: consultancy fees from Daiichi Sankyo. M.E. reports a charitable grant from the AstraZeneca Young Health Programme and personal fees from Prudential, unrelated to this Review. LLL's institution reports receiving grants and/or honoraria for consultancy or lectures from Abbott, AstraZeneca, Boehringer Ingelheim, Novartis, Novo Nordisk, Roche, Sanofi, Servier, and Zuellig Pharma. The other authors declare no competing interests.

## Supporting information


Data S1.
