# Supplementary material for: Obesity-related glomerulopathy: How it happens and future perspectives
Source: Diabet Med. Author manuscript; Available in PMC 2025 May 16. (PMC12080990; doi:10.1111/dme.70042)
Supplement: Supplementary eTable 1 eTable 2 [file EMS204897-supplement-Supplementary_eTable_1_eTable_2.docx]

**Supplementary Materials**

# eTable 1. Pathological differences of nephropathy in individuals with diabetes, hypertension, and obesity

|  | **Obesity-related glomerulopathy ^1^** | **Diabetic nephropathy ^2, 3^** | **Hypertensive nephrosclerosis ^4-6^** |
| --- | --- | --- | --- |
| **Glomerulus** | **Glomerulomegaly**  **Global or focal segmental glomerulosclerosis (perihilar region)**  Low glomerular density  Podocyte injury (detachment and apoptosis)  Glomerulosclerosis  Mesangial expansion | **Glomerular basement membrane thickening**  **Mesangial expansion**  **Kimmelstiel-Wilson lesion** (nodular intercapillary glomerulosclerosis)  **>50 percent global glomerulosclerosis** | Wrinkling of the basement membrane  Collapse of the glomerular tuft  Glomerulosclerosis and tubulointerstitial involvement  Glomerular hypertrophy |
| **Renal vasculature** | Vasodilation of afferent arteriole  Arteriosclerosis | Hyalinosis of the afferent and efferent glomerular arterioles,  interstitial expansion | Myointimal hypertrophy of interlobular arteries  Sclerosis of arterioles,  Hyaline arteriosclerosis |
| **Renal tubule-interstitium** | Tubular hypertrophy/atrophy | Tubular atrophy | Tubular atrophy  Interstitial fibrosis |

Footnotes: The characteristic features associated with each condition are in bold. There is no single pathognomonic feature for hypertensive nephrosclerosis, but a constellation of the features is more suggestive of the condition.

# eTable 2. Emerging biomarkers of obesity-related glomerulopathy

| **Biomarker** | **Study design/ Follow-up (years)** | **Sample size (% males)** | **Study population/ mean age ± SD (years)** | **Mean BMI ± SD (kg/m^2^)** | **Key results** | **Adjustments** |
| --- | --- | --- | --- | --- | --- | --- |
| **Biomarkers of tubular damage** | | | | | | |
| Plasma uromodulin ^7^ | Cross-sectional | 426 (58) | GP (Germany) / 56.8 ± 16.4 | 27.0 ± 5.6 | Plasma uromodulin differentiated non-CKD and CKD in an AUC of 0.924 (95% CI 0.892–0.956). | Age, gender, BMI |
| Urinary uromodulin ^8^ | Prospective / 1.1 | 230 (66) | CKD (Germany) / 60 | 26.0 | Urinary uromodulin in the 1^st^ and 2^nd^ quartile was associated with composite endpoint ESRD/25% eGFR decline of an HR 3.59 (95% CI 1.00-12.99) and HR 5.41 (95% CI 1.44-20.23) | SBP, eGFR, proteinuria, CRP, oral Vit D use, oral phosphate binder use |
| Urinary uromodulin ^9^ | Prospective / 9.5 | 958 (40) | GP (US) / 78.1 ± 4.7 | 26.9 ± 4.7 | Each standard deviation higher urinary uromodulin was associated with a lower risk of 30% eGFR decline of OR 0.77 (95% CI 0.62-0.96) | Age, gender, race, education, clinic site, baseline eGFR, urine ACR, smoking status, pack-years, BMI, DM, SBP, antihypertensive medication use, lipid-lowering medications use, total cholesterol, CRP |
| Serum and urinary NGAL ^10^ | Case-control | 95 (26) | Obesity vs lean controls (Egypt) / 47.4 ± 7.8 | 33.8 vs 22.2 | Individuals with obesity had higher serum and urinary NGAL compared to healthy controls | None |
| Urinary NGAL and KIM-1 ^11^ | Cross-sectional | 117 (35) | T2DM (Brazil) / 58.9 ± 12.8 | 29.8 | uKIM-1 and uNGAL associated with uACR ≥30mg/g with OR 1.056 (95% CI 1.024–1.079) and OR 1.241 (95% CI 1.117–1.380) respectively | HbA1c, LDL cholesterol, fasting glucose, DM pharmacological treatment |
| Plasma KIM-1 ^12^ | Case-control / 8.7 | 1315 (57) | T2DM + CKD vs T2DM (US) / 61 ± 9.3 | 34.1 ± 7.8 | Higher plasma KIM-1 associated with a greater risk of incident ESKD of an HR 1.26 (95% CI 1.14 - 1.40) | Age, sex, race/ethnicity, education, clinical center, systolic BP, diastolic BP, BMI, hs-CRP, hemoglobin A1c, antihypertensive medication use, smoking status, baseline eGFR (ml/min per 1.73 m^2^), and UPCR |
| Urinary NAG ^13^ | Cross-sectional | 57 (61) | GP (Iran) / 56.0 ± 2.5 | - | Urinary NAG was significantly higher (p<0.001) in individuals with microalbuminuria compared to normoalbuminuria. | None |
| Urinary PCX ^14^ | Cross-sectional | 188 (47) | Paediatric (Egypt) / 10.5 ± 1.6 | 26.1 ± 8.7 | ROC curve analysis showed that urinary PCX had 83.3% sensitivity and 74% specificity in the detection of albuminuria (p<0.001) | None |
| Urinary GluAp, AlaAp, Klotho ^15^ | Cross-sectional | - | Male Zucker rats | - | Urinary Klotho showed positive correlations with proteinuria (p=0.0014), urinary GluAp (p<0.0001) and urinary AlaAP (p<0.0001) | None |
| Serum and urinary α-klotho ^16^ | Case-control | 100 (56) | ORG vs CKD vs healthy control vs obesity (China) | 43.4 ± 11.2 | Serum and urinary α-klotho was lowest (p<0.05) in the ORG group compared to CKD, healthy lean and abdominal obesity group | None |
| **Biomarkers of inflammation** | | | | | | |
| Renal tissue MCP and mast cells ^17^ | Cross-sectional | 68 | ORG vs FSGS vs healthy controls / 39.3 ± 9.2 | 32.5 ± 3.8 | Increased number of tubulointerstitial mast cells in ORG individuals compared to FSGS and healthy controls. Renal tissue MCP strongly correlated with mast cell expression in renal tissue. | None |
| Plasma TNFR-1, TNFR-2 ^18^ | Prospective / 12 | 410 (56) | T2DM (US) / 55.9 ± 9.1 | 29.9 ± 6.2 | Plasma TNFR-1 and TNFR-2 had increased risk for incident ESRD for an aHR 9.44 (95% CI 2.83-31.57) and aHR 7.60 (95% CI 2.68-21.57) | AER, eGFR, and HbA_1c_ |
| Serum TNFR-1, TNFR-2, MCP-1, suPAR, YKL-40 ^12^ | Case-control / 8.7 | 1315 (57) | T2DM + CKD vs T2DM (US) / 61 ± 9.3 | 34.1 ± 7.8 | Serum TNFR-1 (β=-0.43; 95% CI -0.81 to –0.05), TNFR-2 (β=-0.53; 95% CI –0.97 to –0.09) and YKL-40 (β=-0.39; 95% CI –0.61 to –0.17) were significantly associated with an annual rate of decline in eGFR | Age, sex, race, education, BP, hs-CRP, BMI, smoking, eGFR, UPCR |
| Plasma SOCS ^19^ | Cross-sectional | 20 (40) | GP (Netherlands) / 59.9 ± 11 | 25.1 ± 3.9 | There is a significant relationship between plasma monocyte SOCS3 and eGFR (r=−0.70, p<0.05) as well as urea (r=0.80, p<0.01). | None |
| Serum ICAM-1 ^20^ | Cross-sectional | 120 (50) | Paediatric (Italy) / 12.6 ± 2.8 | NA | Significant associations were found between serum ICAM-1 and eGFR (β=0.19, p=0.04) and AER (β=0.21, p=0.03). | BMI |
| **Biomarkers of oxidative stress** | | | | | | |
| Plasma ADMA ^21^ | Prospective / 6.8 | 227 (68) | GP (Czechia) / 45.7 ± 12.6 | 25.2 ± 3.8 | An increment of 0.1 µmol/L ADMA increases the risk for disease progression (doubling serum creatinine and/or renal replacement therapy) by OR 1.47 (95% CI 1.12-1.93). | Gender, age, BMI, current smoker, past smoker, GFR, proteinuria, SBP, DBP, pulse pressure, hemoglobin, intact PTH, insulin, glucose, HOMA-IR, total cholesterol, uric acid, hs-CRP, symmetric dimethylarginine (SDMA) |
| Serum MPO ^22^ | Prospective / 5.1 | 3872 (55) | CKD (US) / 58.2 ± 11 | 32.1 ± 7.7 | 1 SD greater log-transformed MPO associated with a higher risk of CKD progression (initiation of RRT or 50% decline in eGFR and eGFR ≤ 15 mL/min/1.73 m^2^) of an HR 1.10 (95% CI 1.01-1.19) | Age, sex, race/ethnicity, BMI, DM, HTN, coronary artery disease, peripheral vascular disease, chronic heart failure, hematocrit, eGFR, 24-hour urine protein excretion, serum albumin level, apolipoprotein L1 risk status, statin use |
| Serum TBARS ^23^ | Prospective cohort/ 3 | 1178 (100) | GP (Japan) / 45 ± 10 | 23.8 ± 2.9 | A statistically significant positive relationship between log-TBARS and BMI (r=0.211, p<0.0001), whereas a negative relationship was shown between log-TBARS and eGFR (r=-0.149, p<0.0001). | Unadjusted |
| **Genomics** | | | | | | |
| Genes *LINC01241*, *LINC00923*, *EXO1* ^24^ | Prospective / 4.1 | 3074 (54) | GP (US) / 58.5 ± 10.9 | 32.2 ± 7.9 | Genes *LINC00923* and *EXO1* increased risk for incident ESRD for an aHR 2.01 (95% CI 1.49-2.71) and 3.92 (95% CI 2.00-7.68), while *LINC01241* reduced risk for an aHR 0.60 (95% CI 0.45-0.81) | Age, sex, and baseline eGFR |
| Genes *SPP1, MMP3, BGLAP, MGP*, and *CYP24A1*  ^25^ | Case-control | 3004 (60) | GP (Spain) / 57.3 ± 12.6 | 28.3 ± 5.1 | 5 SNPs present in genes *SPP1*, *MMP3*, *BGLAP*, *MGP*, and *CYP24A1* combined with clinical risk factor models improved CKD risk detection of AUC 0.824 (95% CI 0.802–0.847) | Age, sex, race, DM, HTN |
| 53 SNP, including gene *UMOD*  ^26^ | Prospective/16.6 | 2301 (42) | GP (Sweden) / 56.0 ± 5.6 | 25.4 ± 3.6 | Compared to the first quartile, a fourth quartile genetic risk score of 53 SNPs is associated with a higher risk for incident CKD (eGFR <60ml/min/1.73m^2^) for an aOR 1.97 (95% CI 1.43-2.70) | Age, sex, baseline eGFR, fasting glucose, BMI, SBP, smoking status (current, former, or never smokers), antihypertensive drugs, follow-up time |
| 22 SNPs, including genes *TPPP* and *FAT1-LINC02374 ^27^* | Prospective / 3.3 | 1738 (63) | CKD (Korea) / 54.9 ± 12.1 | 24.7 ± 6.0 | A genetic risk score of the top 5% 22 SNP compared with the remaining 95% has increased the risk of ESRD for an aHR 1.91 (95% CI 1.29-2.83) | Age, sex, five component principals, baseline eGFR, uACR, DM, SBP, and BMI |
| **Transcriptomics** | | | | | | |
| Renal tissue miR-155 ^28^ | Cross-sectional | - | HFD mice | - | Reduced miR-155 expression significantly reduced microalbuminuria compared with controls without miR-155. | None |
| 5 urinary miRNAs ^29^ | Cross-sectional | - | Obese ZSF1 rats | - | Five urinary mRNA were significantly associated with obese ZSF rats and renin transgenic rats | None |
| Urinary snRNA U6 spliceosomal RNA and has-miR-6124 ^30^ | Cross-sectional | 12 | GP (Korea) / 35.8 | 32.15 | Urinary snRNA U6 spliceosomal RNA and hsa-miR-6124 negatively correlated with eGFR and albuminuria, respectively. | None |
| Urinary miR-205 ^31^ | Prospective / 10 weeks | - | HFD mice | - | Urinary miR-205 detection is associated with downregulation of PTEN in podocytes which is linked to lipid endocytosis and ORG progression | None |
| **Proteomics** | | | | | | |
| 13 plasma proteins ^32^ | Prospective / 14.4 | 9406 (45) | GP (US) / 60.0 ± 5.7 | 28.5 ± 5.4 | 13 distinct proteins significantly associated with composite endpoint ESKD or decline in eGFR by ≥50%, including TNF-receptor superfamily members 1A and 1B, trefoil factor 3, and β-trace protein | Age, sex, race/center, SBP, antihypertension medications, DM, ASCVD, smoking, eGFRcrcys, HDL levels, log-transformed uACR |
| Plasma NT-proBNP, GDF-15  ^33^ | Prospective / 5.8 | 3664 (54) | CKD (US) / 57.8 ± 11.0 | 32.14 ± 7.86 | A high NT-proBNP and GDF-15 showed an increased risk of an HR 1.98 (95% CI 1.51-2.61) and HR 3.46 (95% CI 2.49–4.81) for CKD progression (decline in eGFR by 50% or progression to dialysis or kidney transplant) | Age, sex, race, site, DM, CVD, smoking, 24h urinary protein, eGFR, SBP, BMI, LDL, HDL, ACEi/ARBs, diuretics, beta-blockers, phosphate, PTH, FGF-23, other biomarkers (NT pro-BP, hsTnT, GDF-15, sST-2), year 1 LVM indexed to height, year one ejection fraction |
| Plasma NT-proBNP ^34^ | Prospective / 3.3 | 8005 (64) | GP (US) / 68.0 ± 9 | 29.8 ± 5.7 | A baseline third tertile NT-proBNP compared with first tertile was associated with an increased risk of ≥30% decrease in eGFR for an aOR 1.65 (95% CI 1.05-2.58) | Age, sex, race/ethnicity, randomization arm, baseline CVD, current smoking, BMI, SBP, DBP, antihypertensive meds, diuretics, ACEi/ARB, baseline eGFR, and uACR. |
| Urinary CKD273 ^35^ | Prospective / 2.51 | 1768 (62) | T2DM (Europe) / 61.2 ± 8.7 | 30.1 ± 5 | Compared with a low CKD 273 score, those with a high score (>0.154) had increased risk for uACR >30mg/g for an aHR of 2.48 (95% CI 1.80-3.42) | Age, sex, HbA_1c_, SBP, retinopathy, UACR, eGFR |
| Urinary BMI150 ^36^ | Case-control | 4015 (58) | GP (Germany) / 74.0 ± 10.6 | 25.6 ± 7.9 | BMI150 predicts high BMI and low eGFR (<45ml/min/1.73m^2^) with an AUC of 0.929 (95% CI 0.846–0.975) | None |
| **Metabolomics** | | | | | | |
| 71 serum lipid compounds and 23 serum metabolites ^37^ | Case-control | 50 (51) | Obesity (Spain) / 52.3 ± 12.8 | 42.5 ± 4.7 | Seventy-one serum lipid compounds and 23 serum metabolites significantly differed in individuals with obesity with CKD compared to those without CKD. | None |
| Four serum metabolites ^38^ | Prospective / 8.8 | 962 (61) | CKD (US) / 56 ± 11 | 30.6 ± 6.6 | 4 plasma metabolites were significantly associated with KFRT (dialysis or transplant) and replicated in the MDRD cohort. | Baseline (AASK) or 12-month visit (MDRD) age, sex, trial arms, CVD, h/o smoking, BMI, serum albumin concentration, measured GFR, and in the MDRD study, race, baseline uPCR |
| Plasma pseudouridine, methylimidazoleacetate, homocitrulline ^39^ | Prospective / 6.8 | 1773 (66) | CKD (US) / 58.8 ± 10.8 | 32.2 ± 7.8 | Plasma pseudouridine, methylimidazoleacetate, and homocitrulline were significantly associated with CKD progression (ESRD, eGFR decline 50%) and were replicated in the CRIC and AASK cohort | Age, sex, race, study centre, BMI, SBP, DM, CVD, smoking, alcohol use, APOL1 genotype, log PCR, eGFR |

“GP” were general population observational studies, where no kidney biopsies were available. However, they were selected because of their mean BMI value within the overweight/obesity range of >25 kg/m^2^ in Western populations and >23 kg/m^2^ in Asian populations. “Obesity” populations had 100% obesity at baseline, but also without kidney biopsies. “ORG” represented the populations with kidney biopsy-confirmed ORG with available data for comparisons.

AASK, African American Study of Kidney Disease; ACE, angiotensin-converting enzyme; ADMA, Asymmetric dimethylarginine; ARB, angiotensin receptor blocker; aHR, adjusted hazard ratio; AlaAp, Alanyl aminopeptidase; aOR, adjusted odds ratio; AUC, Area under the curve; BMI, Body mass index; CRIC, Chronic Renal Insufficiency Cohort; CVD, cardiovascular disease; DBP, diastolic blood pressure; DM, Diabetes mellitus; eGFR, estimated glomerular filtration rate; ESRD, End-stage renal disease; GDF-15, growth differentiating factor-15; GluAp, Glutamyl aminopeptidase; GP, general population; HDL, high density lipoprotein; HFD – high fat diet; HOMA-IR, homeostasis model assessment of insulin resistance; hsTnT, high sensitivity troponin-T; HTN, Hypertension; ICAM-1, Intercellular adhesion molecule-1; LDL, low-density lipoprotein; MDRD, Modification of Diet in Renal Disease; miRNA, micro RNA; NGAL, Neutrophil gelatinase-associated lipocalin; KIM-1, Kidney injury molecule-1; MCP-1, Monocyte chemotactic protein-1; MPO, myeloperoxidase; NAD, N-acetyl-beta-D-glucosaminidase; NT-proBNP, N-terminal pro B-type natriuretic peptide; PCX, Podocalyxin ; ROC, receiver operating characteristic; SBP, systolic blood pressure; sST-2, soluble ST2; SD, standard deviation; SOCS, Suppressors of cytokine signaling; snRNA, small nuclear RNA; suPAR, Soluble urokinase-type plasminogen activator receptor; TBARS, Thiobarbituric acid-reacting substance; TNFR-1, Tumour necrosis factor receptor type 1; TNFR-2, Tumour necrosis factor receptor type 1; UACR, urine albumin creatinine ratio; UAE, albumin excretion rate; UPCR, urine protein creatinine ratio

# References

**1**. D'Agati VD, Chagnac A, de Vries AP, et al. Obesity-related glomerulopathy: clinical and pathologic characteristics and pathogenesis. *Nat Rev Nephrol*. 2016;12:453-471.

**2**. Fioretto P, Mauer M. Histopathology of diabetic nephropathy. *Semin Nephrol*. 2007;27:195-207.

**3**. Tervaert TWC, Mooyaart AL, Amann K, et al. Pathologic Classification of Diabetic Nephropathy. *Journal of the American Society of Nephrology*. 2010;21:556-563.

**4**. Tracy RE, Ishii T. What is 'nephrosclerosis'? lessons from the US, Japan, and Mexico. *Nephrol Dial Transplant*. 2000;15:1357-1366.

**5**. Hill GS. Hypertensive nephrosclerosis. *Curr Opin Nephrol Hypertens*. 2008;17:266-270.

**6**. Fogo A, Breyer JA, Smith MC, et al. Accuracy of the diagnosis of hypertensive nephrosclerosis in African Americans: a report from the African American Study of Kidney Disease (AASK) Trial. AASK Pilot Study Investigators. *Kidney Int*. 1997;51:244-252.

**7**. Steubl D, Block M, Herbst V, et al. Plasma Uromodulin Correlates With Kidney Function and Identifies Early Stages in Chronic Kidney Disease Patients. *Medicine (Baltimore)*. 2016;95:e3011.

**8**. Steubl D, Block M, Herbst V, et al. Urinary uromodulin independently predicts endstage renal disease and rapid kidney function decline in a cohort of chronic kidney disease patients. *Medicine (United States)*. 2019;98.

**9**. Garimella PS, Biggs ML, Katz R, et al. Urinary uromodulin, kidney function, and cardiovascular disease in elderly adults. *Kidney Int*. 2015;88:1126-1134.

**10**. Rashad NM, Said NM, Emad G, Gomaa AF, Kadry HM. Assessment of serum and urinary levels of neutrophil gelatinase-associated lipocalin in correlation with albuminuria in nondiabetic obese patients. *The Egyptian Journal of Internal Medicine*. 2019;31:642-651.

**11**. de Carvalho JA, Tatsch E, Hausen BS, et al. Urinary kidney injury molecule-1 and neutrophil gelatinase-associated lipocalin as indicators of tubular damage in normoalbuminuric patients with type 2 diabetes. *Clin Biochem*. 2016;49:232-236.

**12**. Schrauben SJ, Shou H, Zhang X, et al. Association of Multiple Plasma Biomarker Concentrations with Progression of Prevalent Diabetic Kidney Disease: Findings from the Chronic Renal Insufficiency Cohort (CRIC) Study. *J Am Soc Nephrol*. 2021;32:115-126.

**13**. Mohammadi-Karakani A, Asgharzadeh-Haghighi S, Ghazi-Khansari M, Hosseini R. Determination of urinary enzymes as a marker of early renal damage in diabetic patients. *J Clin Lab Anal*. 2007;21:413-417.

**14**. Musa N, Ramzy T, Hamdy A, Arafa N, Hassan M. Assessment of urinary podocalyxin as a marker of glomerular injury in obesity-related kidney disease in children and adolescents with obesity compared to urinary albumin creatinine ratio. *Clin Obes*. 2021;11:e12452.

**15**. Montoro-Molina S, López-Carmona A, Quesada A, et al. Klotho and Aminopeptidases as Early Biomarkers of Renal Injury in Zucker Obese Rats. *Front Physiol*. 2018;9:1599.

**16**. Yang M, Wang G, Rui H, Pei Y, Cheng H, Chen Y. The related study of alpha-klotho and obesity related glomerulonephritis. *Chinese Journal of Nephrology*. 2015;31:140-144.

**17**. Wang X, Chen H, Zhang M, Liu Z. Roles of mast cells and monocyte chemoattractant protein-1 in the renal injury of obesity-related glomerulopathy. *Am J Med Sci*. 2013;346:295-301.

**18**. Niewczas MA, Gohda T, Skupien J, et al. Circulating TNF receptors 1 and 2 predict ESRD in type 2 diabetes. *J Am Soc Nephrol*. 2012;23:507-515.

**19**. Rastmanesh MM, Bluyssen HA, Joles JA, Boer P, Willekes N, Braam B. Increased expression of SOCS3 in monocytes and SOCS1 in lymphocytes correlates with progressive loss of renal function and cardiovascular risk factors in chronic kidney disease. *Eur J Pharmacol*. 2008;593:99-104.

**20**. Marcovecchio ML, de Giorgis T, Di Giovanni I, Chiavaroli V, Chiarelli F, Mohn A. Association between markers of endothelial dysfunction and early signs of renal dysfunction in pediatric obesity and type 1 diabetes. *Pediatr Diabetes*. 2017;18:283-289.

**21**. Fliser D, Kronenberg F, Kielstein JT, et al. Asymmetric dimethylarginine and progression of chronic kidney disease: the mild to moderate kidney disease study. *J Am Soc Nephrol*. 2005;16:2456-2461.

**22**. Correa S, Pena-Esparragoza JK, Scovner KM, Waikar SS, Mc Causland FR. Myeloperoxidase and the Risk of CKD Progression, Cardiovascular Disease, and Death in the Chronic Renal Insufficiency Cohort (CRIC) Study. *Am J Kidney Dis*. 2020;76:32-41.

**23**. Okauchi Y, Kishida K, Funahashi T, et al. Cross-sectional and longitudinal study of association between circulating thiobarbituric acid-reacting substance levels and clinicobiochemical parameters in 1,178 middle-aged Japanese men - the Amagasaki Visceral Fat Study. *Nutr Metab (Lond)*. 2011;8:82.

**24**. Parsa A, Kanetsky PA, Xiao R, et al. Genome-Wide Association of CKD Progression: The Chronic Renal Insufficiency Cohort Study. *Journal of the American Society of Nephrology*. 2017;28:923-934.

**25**. Valls J, Cambray S, Pérez-Guallar C, et al. Association of Candidate Gene Polymorphisms With Chronic Kidney Disease: Results of a Case-Control Analysis in the Nefrona Cohort. *Frontiers in Genetics*. 2019;10.

**26**. Schulz CA, Engström G, Christensson A, Nilsson PM, Melander O, Orho-Melander M. Genetic Predisposition for Renal Dysfunction and Incidence of CKD in the Malmö Diet and Cancer Study. *Kidney Int Rep*. 2019;4:1143-1151.

**27**. Han M, Moon S, Lee S, et al. Novel Genetic Variants Associated with Chronic Kidney Disease Progression. *Journal of the American Society of Nephrology*. 2023;34:857-875.

**28**. Zheng C, Zhang J, Chen X, et al. MicroRNA-155 Mediates Obesity-Induced Renal Inflammation and Dysfunction. *Inflammation*. 2019;42:994-1003.

**29**. Petzuch B, Bénardeau A, Hofmeister L, et al. Urinary miRNA Profiles in Chronic Kidney Injury-Benefits of Extracellular Vesicle Enrichment and miRNAs as Potential Biomarkers for Renal Fibrosis, Glomerular Injury, and Endothelial Dysfunction. *Toxicol Sci*. 2022;187:35-50.

**30**. Choi D, Kim S, Woo J, et al. Weight Change Alters the Small RNA Profile of Urinary Extracellular Vesicles in Obesity. *Obesity Facts*. 2022;15:292-301.

**31**. López-Martínez M, Armengol MP, Pey I, et al. Integrated miRNA-mRNA Analysis Reveals Critical miRNAs and Targets in Diet-Induced Obesity-Related Glomerulopathy. *Int J Mol Sci*. 2024;25.

**32**. Grams ME, Surapaneni A, Chen J, et al. Proteins Associated with Risk of Kidney Function Decline in the General Population. *J Am Soc Nephrol*. 2021;32:2291-2302.

**33**. Bansal N, Zelnick L, Shlipak MG, et al. Cardiac and stress biomarkers and chronic kidney disease progression: The CRIC study. *Clinical Chemistry*. 2019;65:1448-1457.

**34**. Ascher SB, Berry JD, Katz R, et al. Changes in Natriuretic Peptide Levels and Subsequent Kidney Function Decline in SPRINT. *American Journal of Kidney Diseases*. 2023.

**35**. Tofte N, Lindhardt M, Adamova K, et al. Early detection of diabetic kidney disease by urinary proteomics and subsequent intervention with spironolactone to delay progression (PRIORITY): a prospective observational study and embedded randomised placebo-controlled trial. *Lancet Diabetes Endocrinol*. 2020;8:301-312.

**36**. Wendt R, He T, Latosinska A, Siwy J, Mischak H, Beige J. Proteomic characterization of obesity-related nephropathy. *Clinical Kidney Journal*. 2020;13:684-692.

**37**. Lanzon B, Martin-Taboada M, Castro-Alves V, et al. Lipidomic and Metabolomic Signature of Progression of Chronic Kidney Disease in Patients with Severe Obesity. *Metabolites*. 2021;11.

**38**. Luo S, Coresh J, Tin A, et al. Serum Metabolomic Alterations Associated with Proteinuria in CKD. *Clin J Am Soc Nephrol*. 2019;14:342-353.

**39**. Wen D, Zheng Z, Surapaneni A, et al. Metabolite profiling of CKD progression in the chronic renal insufficiency cohort study. *JCI Insight*. 2022;7.
